# PUMAA: A Platform for Accessible Microbiome Analysis in the Undergraduate Classroom

**DOI:** 10.3389/fmicb.2020.584699

**Published:** 2020-10-06

**Authors:** Keith Mitchell, Jiem Ronas, Christopher Dao, Amanda C. Freise, Serghei Mangul, Casey Shapiro, Jordan Moberg Parker

**Affiliations:** ^1^Department of Microbiology, Immunology and Molecular Genetics, University of California, Los Angeles, Los Angeles, CA, United States; ^2^Department of Clinical Pharmacy, School of Pharmacy, University of Southern California, Los Angeles, CA, United States; ^3^Center for Educational Assessment, Center for the Advancement of Teaching, University of California, Los Angeles, Los Angeles, CA, United States

**Keywords:** microbiome, 16S rRNA, software tool, GUI (Graphical User Interface), undergraduate education, curriculum, data visualisation, targeted amplicon sequencing

## Abstract

Improvements in high-throughput sequencing makes targeted amplicon analysis an ideal method for the study of human and environmental microbiomes by undergraduates. Multiple bioinformatics programs are available to process and interpret raw microbial diversity datasets, and the choice of programs to use in curricula is largely determined by student learning goals. Many of the most commonly used microbiome bioinformatics platforms offer end-to-end data processing and data analysis using a command line interface (CLI), but the downside for novice microbiome researchers is the steep learning curve often required. Alternatively, some sequencing providers include processing of raw data and taxonomy assignments as part of their pipelines. This, when coupled with available web-based or graphical user interface (GUI) analysis and visualization tools, eliminates the need for students or instructors to have extensive CLI experience. However, lack of universal data formats can make integration of these tools challenging. For example, tools for upstream and downstream analyses frequently use multiple different data formats which then require writing custom scripts or hours of manual work to make the files compatible. Here, we describe a microbial ecology bioinformatics curriculum that focuses on data analysis, visualization, and statistical reasoning by taking advantage of existing web-based and GUI tools. We created the Program for Unifying Microbiome Analysis Applications (PUMAA), which solves the problem of inconsistent files by formatting the output files from several raw data processing programs to seamlessly transition to a suite of GUI programs for analysis and visualization of microbiome taxonomic and inferred functional profiles. Additionally, we created a series of tutorials to accompany each of the microbiome analysis curricular modules. From pre- and post-course surveys, students in this curriculum self-reported conceptual and confidence gains in bioinformatics and data analysis skills. Students also demonstrated gains in biologically relevant statistical reasoning based on rubric-guided evaluations of open-ended survey questions and the Statistical Reasoning in Biology Concept Inventory. The PUMAA program and associated analysis tutorials enable students and researchers with no computational experience to effectively analyze real microbiome datasets to investigate real-world research questions.

## Introduction

Engaging undergraduates in research has been consistently demonstrated to increase students’ performance, attitudes, and retention in sciences (Lopatto, 2004; Russell et al., 2007; Eagan et al., 2013). In particular, course-based undergraduate research experiences (CUREs) have been touted as an inclusive and scalable model to bring these benefits to a diverse set of student populations (Harrison et al., 2011; Bangera and Brownell, 2014; Corwin et al., 2015; Shapiro et al., 2015; Hanauer et al., 2017). Microbiome research using marker gene metabarcoding is an attractive direction for CUREs, as sample collection is relatively straightforward and advances in sequencing technologies and reduced cost have made the acquisition of marker gene microbiome data easier than ever (Clooney et al., 2016; Jovel et al., 2016). The large microbiome datasets using a combination of marker genes targeting bacteria and archaea (16S), eukaryotes (18S), and fungi (ITS) give students an opportunity to ask a variety of questions ranging from the composition of their own oral microbiome to plant–microbe interactions (Rosenwald et al., 2012; Sanders and Hirsch, 2014; Wang et al., 2015; Weber et al., 2018; Parks et al., 2020; Sewall et al., 2020).

We designed a microbial ecology CURE as part of the interdepartmental Competency-Based Research Laboratory Curriculum at the University of California, Los Angeles (Shapiro et al., 2015). In this two-term (two 10-week quarters) curriculum students work in teams to conduct self-directed research projects, with a focus on developing critical thinking and quantitative skills. Under the umbrella of an instructor designated overarching research question, students in the microbial ecology CURE formulate and test hypotheses about the microbiomes of different environments. The functional profiles of microbial communities are just as important as the taxonomic composition (Langille, 2018), and the questions of “who is there?” and “what are they doing there?” are the guiding questions for the curriculum. In the first wet-lab term they use both cultivation-dependent techniques such as isolating bacteria from the soil and characterizing their functional capabilities, and cultivation-independent techniques such as extraction of environmental DNA (eDNA) for 16S rRNA (16S) sequencing. In the second computer-lab term they use a variety of phylogenetics programs and bioinformatics tools for analysis of microbiome taxonomic community profiles and Piphillin predicted functional profiles (Narayan et al., 2020).

A major challenge for the development of microbiome research for undergraduates is that marker gene amplicon microbiome data provided by sequencing providers requires a number of bioinformatic processing steps before it can be easily analyzed and visualized, a process with which not all instructors or researchers have familiarity (Carey and Papin, 2018; Garcia-Milian et al., 2018). Many of the available end-to-end data analysis packages such as Quantitative Insights Into Microbial Ecology (QIIME/QIIME 2) (Caporaso et al., 2010; Bolyen et al., 2019), mothur, and the Pipeline for Environmental DNA Metabarcoding Analysis (PEMA) (Zafeiropoulos et al., 2020) have steep learning curves, requiring at least some command line interface (CLI) programming skills, or familiarity with R (R: The R Project for Statistical Computing) in the case of phyloseq (McMurdie and Holmes, 2013, 2015) and PEMA, in order to perform data analysis and visualization. Teaching these skills may be outside the scope of the average undergraduate microbiology classroom. Fortunately, there are several microbiome data analysis and visualization tools that do not require command line, such as the Shiny web app ranacapa (Kandlikar et al., 2018) or locally installed programs with graphical user interfaces (GUIs) such as Statistical Analysis of Metagenomic Profiles (STAMP) (Parks and Beiko, 2010; Parks et al., 2014) and Cytoscape (Shannon et al., 2003). These are attractive tools for use in the undergraduate bioinformatics classroom where there is lack of time to devote to the steep learning curve necessary for installation and use of command line programs (Mangul et al., 2017).

Even with the increasing availability of GUI analysis tools, there is still the problem that the data output file formats from QIIME or custom commercial and academic pipelines such as MrDNA (mrdnalab, 2020) and Anacapa (Curd et al., 2019) do not match the data input file formats required for the GUI and web-based analysis and visualization tools. Formatting the different analysis pathway files into a single pipeline is a non-trivial task requiring either running scripts or hours of manual reformatting. To address this problem, we created PUMAA, the Program for Unifying Microbiome Analysis Applications, which takes the output files from QIIME, Anacapa, or MrDNA and reformats them directly for use in downstream GUI or web-based applications for microbiome analysis. Additionally, PUMAA both prepares files for upload to Piphillin for prediction of functional genes from the 16S taxonomy data, and queries the KEGG database to annotate the Piphillin gene predictions (Iwai et al., 2016; Narayan et al., 2020). Inferring functional profiles from 16S rRNA marker genes using programs like PiCRUSt (Langille et al., 2013; Douglas et al., 2020) or Piphillin are accessible options for researchers without the resources to perform full functional metagenomics (Laudadio et al., 2019).

Since classroom time is limited and our curriculum learning objectives focus on microbiome data analysis, visualization, and statistical reasoning rather than learning programming languages, the instructional staff runs the PUMAA program to generate the files necessary for several different GUI or web-based tools and provide them to students. The bioinformatics curriculum is scaffolded such that the students’ progress in their microbiome research from phylogenies of individual bacterial isolates, to simple microbial community qualitative analyses, to quantitative diversity metrics, to statistical analysis of the microbial community profiles. We developed accompanying instructional modules, video tutorials, and a lab manual to teach students both the theory behind the analysis tools and the skills needed for visualizing and performing biostatistical methods on the data. The key tools and tutorials include inferring phylogenetic trees, analyzing community profiles and diversity metrics using Microsoft Excel pivot tables and ranacapa, statistical analysis of taxonomic and inferred functional profiles using STAMP, and using KEGG to assign functions to genes.

The curriculum was assessed using entry/exit surveys designed to gauge the students’ confidence in integrating computational analysis with microbiology, and the Statistical Reasoning in Biology Concept Inventory (SRBCI) (Deane et al., 2016). Analysis of entry and exit surveys saw an increase in students’ self-reported conceptual understanding and confidence levels in using the analysis tools, as well as improved competencies with biostatistics as demonstrated by improvement in the SRBCI post-test. The PUMAA program and associated instructional materials provide a scaffolded learning experience for undergraduate students and make microbiome bioinformatics analyses accessible to novice researchers.

## PUMAA – Program for Unifying Microbiome Analysis Applications Overview

Analyzing metabarcoded microbiome data is a complex multi-step process. Next-generation sequencing produces a variety of data files, which then need to be processed and quality checked before assigning taxonomic profiles (Zhang, 2016; Almeida et al., 2018). Most sequencing providers include basic bioinformatic processing in their pipelines, and provide taxonomic abundance tables and sequence FASTA files along with the raw data. These files can be then used in downstream analysis and visualization applications. However, each taxonomic assignment platform and analysis or visualization tool may have different data input and output formats that need to be reconciled, or have significant data pre-processing steps that need to occur before the various analyses can be performed.

**FIGURE 1:**
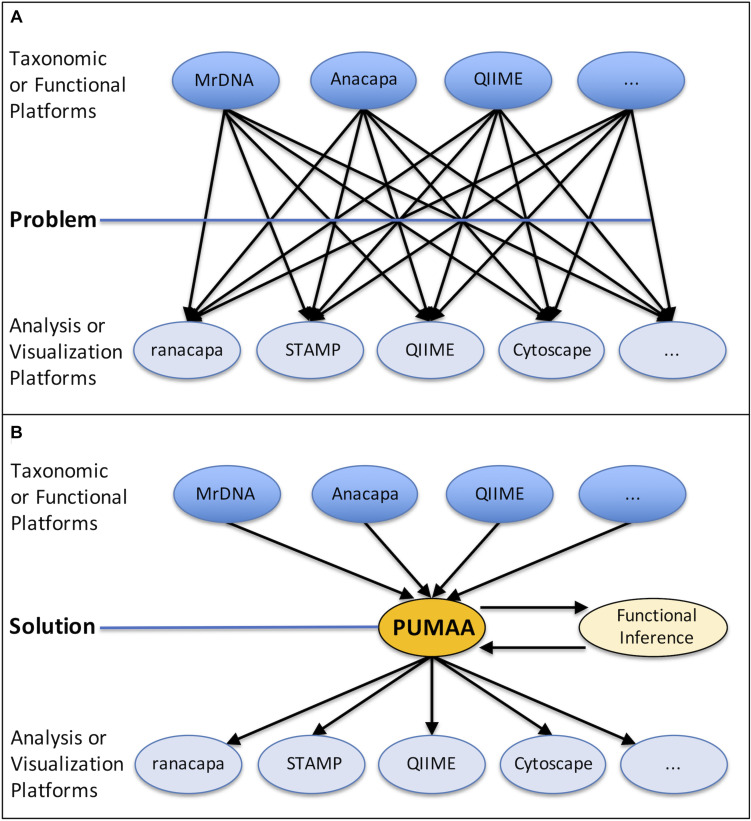
The problem presented and the PUMAA solution. **(A)** The current problem is lack of unification of outputs from different taxonomic identification or functional inference platforms (MrDNA, Anacapa, QIIME, etc.) and the input data required by prospective analysis and visualization tools (ranacapa, STAMP, QIIME, Cytoscape, etc.). **(B)** PUMAA is a streamlined pipeline unifying the output files from multiple platforms and converting them to the input files necessary for varied analysis and visualization tools.

Some sequencing providers, such as MrDNA (mrdnalab, 2020), produce taxonomy abundance tables that must be rearranged in order to be compatible with most visualization programs, but even for those that are in the right general format, many tools have specific formatting requirements. For example, the STAMP tool enforces a “strict hierarchy” requirement where no classification of taxonomy can exist at a lower level than one which was left unclassified. The following classification, from phylum to species: “Proteobacteria, Gammaproteobacteria, Enterobacteriales, unclassified, *Escherichia*, unclassified,” will produce errors in STAMP because the family is unclassified even though the genus is classified. In addition, STAMP requires that all unclassified columns must be labeled so and cannot be left blank. Another tool, Cytoscape, requires that each sample identification and taxonomic identification be a unique row where the weight corresponds to the quantity of the given instance in order to create a network type visualization. Web server-based programs such as Piphillin (Iwai et al., 2016) may have file size upload limitations, necessitating sub-setting of the data. These formatting and processing steps need to be carried out independently on the taxonomy or functional data for each of the desired analysis and visualization platforms (Figure 1A).

PUMAA, the Program for Unifying Microbiome Analysis Applications, provides the solution to these problems by integrating all of the formatting and pre-processing steps required for the platforms and tools discussed here into a single unified protocol with an easy installation procedure (Figure 1B). In addition, PUMAA is easily expandable as it provides the ability to add a new analysis tool or taxonomic ID platform with one added operation. The PUMAA protocol unifies existing data analysis and visualization tools by formatting common amplicon (16S/18S/ITS) taxonomic data outputs from a variety of sources to be compatible with the input formats required for multiple basic and advanced microbiome analysis tools. Additionally, PUMAA integrates Piphillin inferred functional microbiome composition from the 16S taxonomy data. PUMAA provides both a CLI as well as a GUI to accommodate a spectrum of potential users. A CLI version is implemented to allow users with UNIX experience, or those who are interested in learning, to customize their analysis and build upon/automate the provided scripts (Mangul et al., 2017). The GUI is ideal for novice microbiome researchers with little experience on UNIX based systems, who are interested in quickly visualizing their microbiome marker gene amplicon data. Initial installation of the GUI does require running a small set of terminal installation commands, but subsequent usage is straightforward.

### PUMAA Supports Input From Various Microbiome Data Pipelines

**FIGURE 2:**
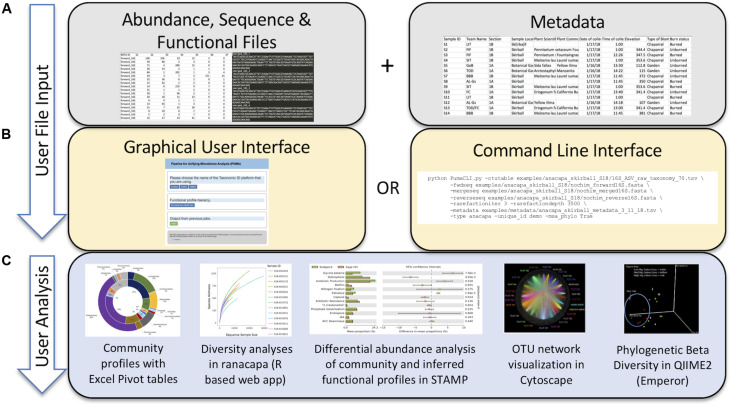
Protocol of the PUMAA software. **(A)** The first panel as part of the “User File Input” displays the simple protocol to be performed by the user such as uploading metadata and various data formats of supported operational taxonomic unit, sequence, and functional file types. **(B)** The second panel as part of the “User File Input” displays the two forms of user interaction with PUMAA, through the GUI and CLI, which will enable community and functional profile analysis. **(C)** “User Analysis” shows the possible platforms for visualizing community/functional composition data enabled by user input such as STAMP, Excel, QIIME 2, and Cytoscape.

Currently PUMAA supports three microbiome raw data processing platforms and/or services: MrDNA, Anacapa, and QIIME 2 (Bolyen et al., 2019; Curd et al., 2019; mrdnalab, 2020). PUMAA formats the taxonomic abundance tables and sequence files created by these platforms for any marker gene amplicons, including 16S, 18S, ITS, and others, for downstream analysis and visualization (Figure 2).

#### MrDNA

MrDNA is a commercial full-service next generation sequencing provider that offers 16S, 18S, and ITS amplicon sequencing on a variety of platforms. Regardless of the sequencing platform, MrDNA provides free comprehensive taxonomic analysis in addition to raw data processing using their proprietary pipeline. The pipeline generates operational taxonomic unit (OTU) abundance tables with taxonomic identities and representative FASTA sequence files at each taxonomic level (kingdom, phylum, class, order, family, genus, species).

#### Anacapa

Anacapa is a software tool kit developed to process environmental DNA (eDNA) sequence data and assign taxonomy data for six marker genes targeting bacteria, archaea, algae, fungi, protozoa, plants, and animals (Curd et al., 2019). Anacapa creates a custom reference library for marker genes, generates amplicon sequence variants (ASV), and assigns taxonomies at each taxonomic level (domain, phylum, class, order, family, genus, species). ASVs have been proposed as a finer resolution replacement for OTU clustering based on sequence similarity (Callahan et al., 2017). Anacapa output includes a detailed taxonomy table with sequences and abundances for each ASV, as well as tables with taxonomies summarized at various percent confidence intervals.

#### QIIME

QIIME is a powerful and widely adopted package for processing microbiome data, from raw sequences through taxonomy and data visualization. Tutorials and published protocols are available to walk users through standard data processing (Kuczynski et al., 2011), but the scope of QIIME may be daunting for novice users, even with the availability of the QIIME 2 Studio graphical interface (Bolyen et al., 2019). It also remains difficult to convert to other analysis/visualization platforms since QIIME provides users with OTU files and sequence files in the ‘.qza’ format, which is unique to its platform.

### PUMAA Supports Piphillin for Inferred Functional Profile Analysis

PUMAA formats taxonomic abundance (OTU or ASV) tables and representative sequence files for prediction of metagenomic content by Piphillin, which uses nearest-neighbor matching of 16S rRNA amplicons and full genomes (Iwai et al., 2016). Piphillin has the added benefits of a web interface and the ability to use any standard abundance table and representative sequence FASTA file, rather than relying on taxonomic assignments assigned from a specific reference phylogenetic tree, as in PiCRUSt (Langille et al., 2013). PiCRUSt2 has an extended database of reference genomes and broader compatibility, but still requires use of the command line for implementation (Douglas et al., 2020). A drawback to Piphillin is the 10 MB limit placed on uploaded file sizes in the web version. PUMAA addresses this by producing subset abundance and FASTA files that comply with these limits. The subset files are uploaded to the Piphillin server^1^, and reference database and percent identity cutoffs are chosen [PUMAA currently only supports KEGG (Kanehisa, 2000; Kanehisa et al., 2004)], then results are emailed to the user as compressed.tar files. The other drawback to Piphillin is that it provides abundance tables for all predicted genes and pathways (identified by K and KO numbers), but not the associated annotations to assign biological information to the K/KO numbers. To address this, the PUMAA inferred function protocol also performs queries to the KEGG database in order to properly annotate the genes and pathways returned by Piphillin. Prior to PUMAA, this annotation process required command-line experience or labor-intensive manual curation.

### PUMAA Supports a Variety of Analysis and Visualization Platforms

There are a wide variety of research questions that can be addressed using amplicon microbiome data, and the methods used for data analysis and visualization will vary based on the needs of the researcher. PUMAA focuses on processing and formatting user data to be compatible with a suite of readily available web-based or GUI data analysis and visualization tools. Using the PUMAA supported tools, researchers can explore data and test hypotheses by linking groups of samples or environmental parameters, otherwise known as metadata, to diversity metrics, community composition, and inferred functional profiles.

We have integrated PUMAA into a broad range of research analysis options (from simple to advanced) and visualization types (from bar charts to network analyses). In addition, PUMAA has options to complete data processing such as rarefaction subsampling to normalize for variation in sequence numbers between samples (McMurdie and Holmes, 2014; Willis, 2019), multiple sequence alignment (MSA) using MUSCLE (Edgar, 2004), and inference of phylogenetic trees using FastTree (Price et al., 2010).

#### Microsoft Excel

Microsoft Excel pivot tables are an easy way to begin to summarize the massive amounts of data in taxonomic abundance tables for visualizations of the overall community profile of different samples at different taxonomic levels (i.e., kingdom/domain, phylum, class, order, family, genus, species). Excel can also be easily used to make simple (non-statistical) comparisons of sample abundances at different taxonomic levels.

#### ranacapa

ranacapa (Kandlikar et al., 2018) is a user-friendly Shiny web application designed to explore biodiversity using environmental DNA metabarcoding data. It includes interactive visualizations and brief explanations of sequencing depth, alpha and beta diversity, and taxonomy distribution analyses such as bar plots and heatmaps. ranacapa was developed as an extension of the Anacapa toolkit (Curd et al., 2019), but can prove slightly difficult to access from other taxonomic identification platforms, like that of MrDNA.

#### STAMP (Statistical Analysis of Metagenomic Profiles)

STAMP (Parks et al., 2014) is a downloadable graphical interface that can quickly generate publication-quality graphics for differential abundance analysis of either taxonomy or functional pathway data without the need to write code or use command-line interface. STAMP supports parametric and nonparametric statistical hypothesis testing for two-sample, two-group, and multiple-group comparisons. It emphasizes the use of effect size and confidence intervals in assessing biological relevance, and supports a variety of visualizations, including heatmaps, PCA plots, extended error bar plots, box plots, and bar plots.

#### QIIME 2 (Quantitative Insights Into Microbial Ecology)

QIIME 2 (Bolyen et al., 2019) provides numerous interactive and advanced data visualization tools and plugins for evaluation of metagenomic profiles (Caporaso et al., 2010; Kuczynski et al., 2011). Although QIIME can be used for end-to-end data analysis, some researchers may receive data processed by other platforms (e.g., MrDNA or Anacapa) and wish to feed the data back into the QIIME pipeline for analysis.

#### Cytoscape

Cytoscape (Kohl et al., 2011) is a unique open-source locally downloadable tool that enables the visualization of networks between community and functional profiles. Basic network analysis and visualization can be performed with the core distribution, with many additional features available as Cytoscape Apps.

### Methods – PUMAA Protocol

#### Overview

The user executes a single script for both the GUI and CLI versions in order to execute the program. The PUMAA protocol consists of two key parts: (1) Production of all files for taxonomic community analysis, and (2) production of all files required for inferred functional analysis. PUMAA solves the problem of going from any of the taxonomic identification platforms to the multitude of visualization and analysis tools available by enforcing standardized files as part of the unification process. The user first obtains input files from one of the three supported pipelines (MrDNA, Anacapa, or QIIME2), identifies the metadata necessary for identifying and comparing samples (Figure 2A), and chooses to run PUMAA through either the GUI or CLI (Figure 2B). PUMAA verifies that the metadata sample IDs match the input data, then produces output files that can be used for a variety of analysis platforms (Figure 2C).

#### Protocol: PUMAA Installation and Requirements

PUMAA is freely available under the Apache-2.0 license at https://github.com/keithgmitchell/PUMAA and is supported by MacOSX and Linux; in addition, PUMAA works on Windows machines after installing the Linux subsystem Comprehensive installation instructions are provided on the Github page. Given software install is handled using conda, all versions of MacOSX and Linux that support the conda environment management software are viable options for usage and make for consistent and user-friendly install (Mangul et al., 2019). Issues or questions with the software can be submitted using the github issues feature: https://github.com/keithgmitchell/PUMAA/issues.

PUMAA is written in Python and the application’s GUI is written using the Django web framework running locally. The example datasets all run on a laptop and use <1GB of memory when the MSA and Phylogenetic tree production is set as false. The QIIME 2 and MrDNA datasets run on a laptop and use <1GB of memory when the MSA and Phylogenetic tree production is set as true. The Anacapa dataset was unsuccessful on a laptop with 16GB RAM and was evaluated using a high-performance computing (HPC) cluster with 32GB of RAM and 3 h of runtime. Therefore, to produce a MSA and phylogenetic tree for datasets of this size, access to an HPC cluster, experience with CLI, and experience running jobs on HPC clusters may be required (Table 1).

**TABLE 1:** Dataset size, runtime, and memory usage with no rarefaction performed across the three example datasets.

Dataset	Dataset size (ASV/OTU count *10,000)	Fasta file size (MB)	Runtime (minutes)	FastTree/MAFFT peak memory usage (GB)	Python memory usage (GB)
MrDNA examples	0.3229	0.868	0.0778	0.207	0.02
QIIME 2 examples	0.0759	0.115	0.00517	0.044	0.02
Anacapa examples	3.6	1.789	1.24	12	0.075

#### Protocol: PUMAA Verifies Metadata

The user uploads their metadata describing the samples, taxonomy abundance (OTU or ASV) table and sequences from any given supported platform. The first part of the PUMAA protocol verifies the metadata and the taxonomy table to be sure the two files have consistent, alphanumeric sample identifiers which are unique compared to other forms of metadata validation (Rideout et al., 2016). This is a critical step as identifiable metadata is necessary for many downstream analysis steps, and some tools limit the types of characters accepted in the sample identifiers (e.g., underscores, but not periods, are acceptable in sample IDs in ranacapa).

#### Protocol: PUMAA Produces Files for Community Profile Analysis

PUMAA performs a variety of functions on the taxonomic abundance and sequence files in order to support the suite of tools discussed above. These functions include optional sample rarefaction at a user defined depth and number of iterations (max = 10) (Weiss et al., 2017), multiple sequence alignment by MAFFT (Katoh and Standley, 2013), phylogenetic tree construction via FastTree 2 (Price et al., 2010), and file formatting and annotation for ranacapa, STAMP, QIIME 2, Piphillin, and Excel. The protocol produces files for community profile analysis in the folder ‘output,’ or some other specified directory as an argument in the CLI. The output folder contains time-stamped subfolders for each PUMAA run, each containing subfolders with ready-to-run files for community profile analyses in Microsoft Excel, STAMP, ranacapa, and Cytoscape. In addition, pre-processed feature table (taxonomy), metadata, and phylogenetic tree files are created that can be imported directly into the QIIME 2 pre-configured virtual machine. A variety of analyses such as alpha- and beta-diversity can be performed in QIIME 2, as well as principal component analysis based on phylogenetic diversity metrics.

#### Protocol: PUMAA Produces Files for Inferred Functional Profile Analysis

The PUMAA protocol consists of three steps necessary for the generation and visualization of inferred functional profiles. The first step is automatically performed at the same time as the generation of the community profile analysis files. PUMAA creates a “piphillin” subfolder in the time-stamped output subfolder. This folder contains the original data formatted as a ‘phiphillinotu.csv’ taxonomic abundance table and a ‘phiphillinseqs.fasta’ representative sequence file. If the FASTA file exceeds the file size limit of 10 MB enforced by the Piphillin server, PUMAA subsamples the data into the number of necessary file sets of ‘.fasta’ and ‘.csv’ files (e.g., piphillinseqs1.fasta; piphillinseqs2.fasta; piphillinotu.csv1.csv; piphillinotu.csv2.csv). Second, each of the sets of Piphillin files in the output directory are uploaded to the Piphillin functional inference web server, which returns ‘.tar’ files to the user via email.

Finally, the ‘.tar’ files can then be run directly in the PUMAA protocol, which produces files for functional analysis that can be visualized using many of the same tools used for community profile analysis, including STAMP, Excel, and QIIME 2. Importantly, the PUMAA protocol also performs queries to the KEGG database using the KEGG genes to pathway API in order to properly annotate the Piphillin gene estimations (Kawashima et al., 2003). The BRITE hierarchy file of the KEGG database is downloaded and used to evaluate the functional hierarchy based on Piphillin pathway estimations. This ensures that estimated gene expression levels and hierarchy levels are inferred using the actively updated information. Annotating the genes and pathway expression from Piphillin is necessary when producing data visualizations with informative identifiers, and greatly reduces the need for manual querying of KEGG.

PUMAA produces a timestamped output subfolder for the functional profile files, including a gene description and functional hierarchy file designated for use in STAMP and Excel. This file contains annotated gene names and functional pathways, as opposed to just “K number” identifiers, and vastly increases the efficiency and ease of data analysis and visualization. PUMAA also produces weighted functional network files for usage in Cytoscape, which is a platform for visualizing important gene networks between samples.

#### Sample Data

The sample data used here and in the tutorials was generated by UCLA students in the winter and spring quarters of 2018, where they investigated the effect on rhizosphere microbial communities following the Skirball wildfire of December 2017 (Skirball Fire, 2020). Sample collection kits and sample sequencing were provided by the California Environmental DNA (CALeDNA) program, a community science initiative monitoring California’s biodiversity through eDNA (Meyer et al., 2019), and the 16S sequences were processed using the Anacapa toolkit (Curd et al., 2019). The sample data for QIIME 2 is the same as the “moving pictures” human microbiome example dataset available on the QIIME 2 website^2^.

### Results

#### PUMAA Input and Output Files

The PUMAA pipeline creates output files formatted specifically for the needed input files for each of the data analysis and visualization platforms described in Supplementary Table 1.

## PUMAA – Curriculum Overview

The Microbiology, Immunology, and Molecular Genetics (MIMG) undergraduate degree program at UCLA requires the completion of a two-quarter authentic research experience. An option to fulfill this requirement is to take the MIMG 109AL/BL: Research Immersion Laboratory in Microbiology series. This laboratory series is designed to prepare its students with the proper background and training to work in microbiology research, and has been demonstrated to improve their critical thinking and research skills as part of the life science curriculum (Shapiro et al., 2015). The 109AL/BL laboratory curriculum is discovery-based and driven by student-generated hypotheses tested using both cultivation-dependent and cultivation-independent techniques. The first term emphasizes experimental design and isolation of bacteria in a wet lab environment, and the second term focuses on the analysis of 16S sequencing data from individual isolates and 16S rRNA microbial community profiles. Students work in teams to conduct an original research project within the context of an overarching research question for the microbial ecology course, focusing on the interactions between plants and soil-associated bacteria. Recent course projects have involved collaborations with researchers at UCLA and beyond studying plant–microbe interactions in California grasslands (Kandlikar et al., 2020), analysis of the soil microbial communities of a Los Angeles urban farm (St. Clair et al., 2020), and a longitudinal study on the recovery of soil microbial communities following the 2017 Skirball fire in Los Angeles, CA, United States. The Skirball fire project was conducted in conjunction with the California Environmental DNA (CALeDNA) program’s efforts to catalog California’s biodiversity (Meyer et al., 2019).

In order for the MIMG 109AL/BL lab series to respond to the need for more computationally minded scientists (Bialek and Botstein, 2004; Campbell et al., 2007; Brewer and Smith, 2011), it was necessary to introduce new modules and tutorials that would sufficiently integrate bioinformatics and statistics with biology in ways that aspiring undergraduate researchers can comprehend (Aikens and Dolan, 2014). We created a comprehensive set of step-by-step tutorials (documents, presentations, and videos) designed to provide students with the necessary theory and skills to use the GUI analysis and visualization tools described in Section 2.3 (Excel, ranacapa, and STAMP), as well as the theory behind inference of metagenomic functional profiles using Piphillin. Although not a biostatistics course, the PUMAA-associated curriculum allows these students to learn about the computational tools available to researchers and the importance of integrating their knowledge of microbiology with statistical and quantitative support.

All tutorials are publicly available at https://sites.google.com/g.ucla.edu/pumaa/home.

### First Term – Sample Collection and Bacterial Isolation/Characterization

The first term of the curriculum takes place in the wet lab and closely follows the cultivation-dependent experiments described in units 1–4 of the “I, Microbiologist” (Sanders and Miller, 2010) course textbook and lab manual. In brief, students collect bulk soil and decide on enrichment strategies for isolation of bacteria related to their research questions (e.g., antibiotic production and resistance or plant growth-promoting properties). Students then perform phenotypic characterization of bacterial isolates and 16S rRNA PCR and sequencing. In addition to collecting bulk soil for cultivation-dependent experiments, students also collect separate soil samples for environmental DNA (eDNA) extraction and 16S rRNA high-throughput sequencing for bacterial community profile analysis.

### Second Term – Bioinformatics Analysis of 16S rRNA Genes Using PUMAA

**FIGURE 3:**
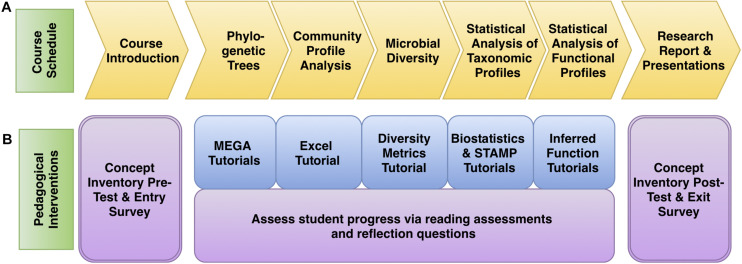
Microbiome analysis course schedule with pedagogical interventions. **(A)** The progress of the course followed the concept goals as outlined in yellow. **(B)** The pedagogical interventions are described, with tutorials in blue and assessment materials in purple.

In the second term, students use bioinformatics to interpret, expand, or refine 16S rRNA gene datasets generated in MIMG 109AL. Students generate 16S rRNA phylogenetic trees to assign taxonomic identities to their isolates and use statistical tools to make comparisons of the microbial communities from different environments. The course is divided into five Core Concept Modules. The first module (Phylogenetic Trees) concludes the analysis of bacterial isolates, and the other four modules focus on microbiome data analysis and visualization using the PUMAA output files: Community Profiles, Diversity Metrics, Statistical Analysis of Taxonomic Profiles, and Inferring Metagenomic Functional Profiles (Figure 3A). Students could also elect to perform optional advanced independent analysis on their data using QIIME or Cytoscape. Each of the modules includes written and/or video tutorials and was assessed with a combination of reading assessments and reflection questions (Figure 3B). This bioinformatics course was assessed using pre- and post-course concept inventories and surveys. Learning objectives, activities, and tutorials for each of the Core Concept Modules are outlined in Supplementary File 1.

### Curriculum Assessment Methods

#### Study Sample

The study sample consisted of six cohorts of junior and senior level students who enrolled in MIMG 109BL (Advanced Research in Microbiology) in Spring 2016, Spring 2017, Winter 2018, Spring 2018, Winter 2019, and Spring 2019. This yielded an initial population of 143 students. Table 2 provides a summary of demographic characteristics for these students. Instructor J.M.P. taught the spring cohorts and instructor A.F. taught the winter cohorts. Prerequisites for enrollment in MIMG 109BL included MIMG 109AL (Research Immersion in Microbiology) and either Statistics 13 (Introduction to Statistical Methods for Life and Health Sciences) or Life Sciences 40 (Statistics of Biological Systems).

**TABLE 2:** Study sample demographics.

	Number of students (*N*)	Percent of students (%)
Female	81	56.6%
Transfer student^a^	34	23.8%
URM^b^	34	23.8%
Pell Grant Recipient^c^	53	37.1%
Total	143	100%

*Academic terms: Spring 2016, Spring 2017, Winter 2018, Spring 2018, Winter 2019, Spring 2019. ^*a*^Transfer to UCLA, usually from a 2-year institution. ^*b*^Under-Represented Minority (URM) students include American Indian, Native American, Black Non-Hispanic, and Hispanic students. ^*c*^Received Pell Grant for one or more terms while enrolled at UCLA; Pell Grant Recipient is a proxy for low socioeconomic status.*

#### Assessment Data Collection and Analyses

The study utilized two sources of data: student assignments and self-report surveys. Data collected included qualitative and quantitative measures. UCLA’s Institutional Review Board (IRB) gave approval to work with human subjects on all aspects of the assessment (IRB #10-000904).

#### Administration of Self-Report Surveys

Two self-report surveys were administered to all students in the course. Surveys included a broad collection of open- and closed-ended questions, some developed by the instructors and evaluation team. Students were given the entry survey at the start of the second term and asked to indicate how well they thought they understood key learning goals related to data analysis and their confidence in their ability to analyze data using various visualization plots. The exit survey was completed at the end of the term and had matched questions to the first survey, as well as additional survey questions asking them to assess the quality and usefulness of the tutorials and instructional materials. Both surveys also included open-ended content-related questions. The surveys were piloted in 2016 and 2017 and were given to students anonymously through the course management system as low-stakes (completion points) assessments to increase response rate and reduce response bias (Furnham, 1986). Starting in Winter 2018, these items were added to a comprehensive curricular assessment plan administered electronically by external evaluators (see Shapiro et al., 2015) for details on survey data collection). Of the 143 students who took the course between Spring 2016 and Spring 2019, 141 completed the first survey (98.6% response rate) and 132 completed the second survey (92.3% response rate). The surveys are available as Supplementary File 2.

#### Administration of SRBCI Concept Inventory

The Statistical Reasoning in Biology Concept Inventory (SRBCI) is a series of multiple-choice questions to test students on concepts including statistical significance, basic graph/trend interpretation, and assessing hypotheses based on results (Deane et al., 2016). The twelve questions on the SRBCI pre- and post-tests are designed to identify students’ common misconceptions in statistical analysis and track their learning progress as a result of the pedagogical interventions. The concept inventory was administered as an anonymous low-stakes (ungraded) in-class activity at the start and end of the second term to the first two cohorts of students in Spring 2016 and Spring 2017. The study design, intended to gauge authentic learning gains across the curriculum by reducing “math anxiety” (Ashcraft and Moore, 2009), necessarily resulted in the inability to assess individual student learning gains using this metric. The pre-test and post-test were administered to a total of 52 and 50 students, respectively. Statistical reasoning gains between the pre-test and post-test groups were assessed using descriptive and Mann–Whitney nonparametric tests to account for variations in sample size.

#### Analyses of Closed-Ended Quantitative Survey Data

The closed-ended survey questions quantitatively ranked the students’ agreement with a statement or confidence with a certain concept using a five-point Likert scale ranging from “Not at all” to “Very well/Very confident.” Scores for matched questions were averaged across all participants to compare results from the Entry and Exit Surveys. Survey items asking students about the usefulness of learning activities were rated on a five-point Likert scale where 1 = “Don’t remember,” 2 = “Not useful,” 3 = “Somewhat useful,” 4 = “Very useful,” and 5 = “Essential.” Descriptive analyses of matched pre/post-survey close-ended items were conducted to explore students’ change in self-reported confidence and changes in their self-reported levels of understanding. To test for statistical differences between the overall means of the Entry and Exit Survey items, descriptive and Mann–Whitney nonparametric tests were performed on the combined survey data from all cohorts to account for variations in sample size. Because the responses for the Spring 2016 and Spring 2017 surveys were anonymous, we were unable to pair the data by student. Wilcoxon signed ranks (paired nonparametric) tests were conducted on just the surveys administered by the external evaluators from Winter 2018 to Spring 2019, in order to see if there were differences between the all the data and the matched data. Since both sets of tests were significant, we were confident in using the aggregated data and the Mann–Whitney nonparametric tests to report our results.

#### Analyses of Open-Ended Qualitative Survey Data

Open-ended questions related to course content were included in the Entry and Exit surveys, allowing students to respond in their own words. Of particular interest was a question that asked students to describe the relationship between *p*-value (statistical significance) and effect size (biological significance). A 4-point rubric assessing students’ level of proficiency with statistical concepts was used to gather direct evidence of student learning gains (Supplementary File 3). Student responses to open-ended questions were scored on a scale of 1 point = no familiarity (i.e., students indicated that they are not familiar with the concept), and 2–4 points for novice, intermediate, and advanced proficiency, respectively. Responses left blank were unscored. All student responses (both pre and post) were randomized and pooled by the external evaluator, then provided to the raters. The rubric was developed and refined by J.R., A.F., and J.M.P. through iterative rounds of scoring a subset of sample responses followed by consensus discussion. All responses were scored independently by all three raters, and interrater reliability (IRR) as determined by Randolph’s free-marginal multirater kappa, was 0.49 (61.8% overall agreement) indicating moderate agreement. To account for the IRR variations, the median score for each response was used to assess whether pre-post gains were statistically significant between the groups using both the Mann–Whitney nonparametric test and a *t*-test.

### Curriculum Assessment Results

#### Conceptual and Confidence Gains From Self-Reported Surveys

**FIGURE 4:**
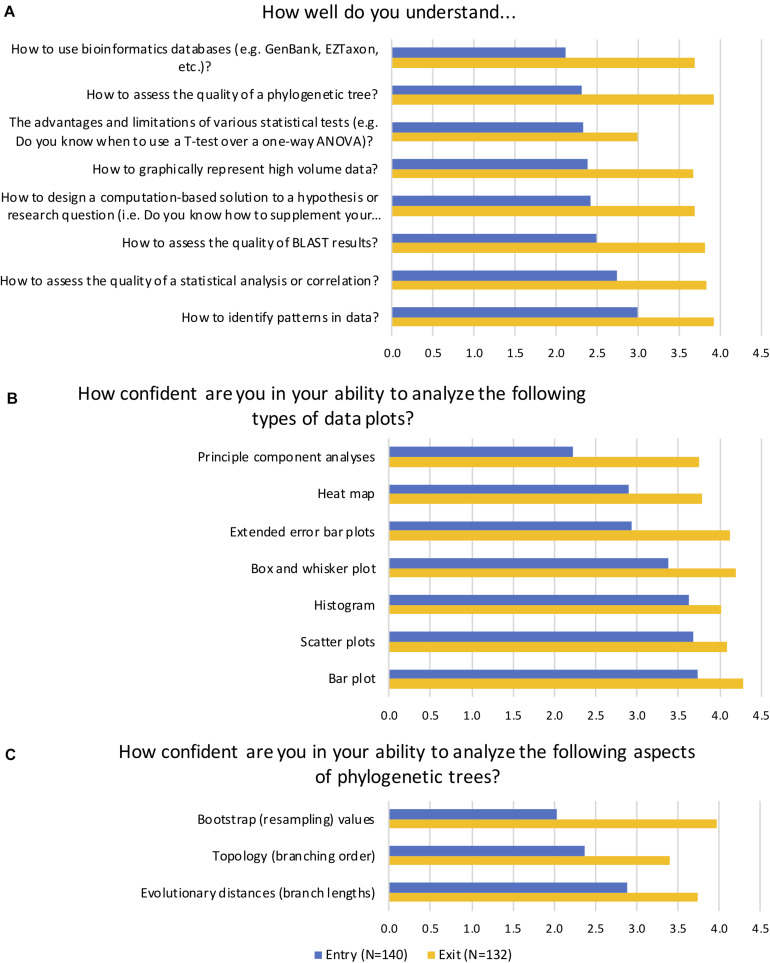
Average ranked responses to selected entry and exit survey questions. In self-reported survey questions, students were asked to indicate **(A)** their level of understanding of key learning goals, **(B)** their confidence in their ability to analyze common data plots, and **(C)** their confidence in their ability to analyze aspects of phylogenetic trees. Average scores on a five-point Likert scale are reported for matched questions. A score of 1 = Not at all, 2 = Very little/Not very, 3 = Fairly well/confident, 4 = Quite well/confident, and 5 = Very well/confident. Students reported significant gains in their understanding and confidence in all categories (*p* < 0.001).

We wanted to assess if students would be able to formulate and statistically test hypotheses linking environmental parameters (metadata) to diversity metrics, community composition, and inferred functional profiles. Students were assessed using entry/exit surveys designed to gauge the students’ comfort with integrating computational analysis with microbiology. At the beginning of the term the students reported, on average, “very little” understanding of key learning objectives such as how to use and assess the results of bioinformatics databases, and which statistical tests to use and how to interpret them (Figure 4A). By the end of the term students reported they understood these concepts on average “fairly well” to “quite well,” a statistically significant change based on Mann–Whitney nonparametric tests for all measures (*p* < 0.001). Of note, students were generally less confident of their understanding of “the advantages and limitations of various statistical tests (e.g., Do you know when to use a *T*-test over a one-way ANOVA)?” at the end of the term. This result was somewhat to be expected because the statistical analysis tool they used, STAMP, aims to promote best practices by suggesting a statistical hypothesis test based on the input data (Parks and Beiko, 2010). Therefore, students had limited practice with this particular skill.

In addition to performing statistical tests, STAMP generates a variety of data visualization plots, and we wanted to assess how confident students were in their ability to analyze these plots (Figure 4B). Mann–Whitney results indicated a statistically significant change in students’ self-reported levels of confidence (*p* < 0.001). Specifically, at the start of the term students reported being “fairly” to “quite” confident in their ability to analyze common plots such as scatter plots, bar plots, and histograms. They had much less confidence, however, in their ability to interpret principal component analysis (PCA), heat maps, and extended error bar plots. By the end of the term they were “quite confident” on average in their ability to analyze most of the plots, and had dramatically improved their confidence in PCA, heat map analyses, and extended error bar plots. Another key learning objective of the course was the ability to interpret phylogenetic trees and analyze their statistical support (Figure 4C). At the start of the term, students reported being “not very” confident in their ability to assess bootstrap or resampling values, which are an indication of the of statistical confidence in a clade (Efron et al., 1996), and “not very” to “fairly” confident in their ability to interpret topology and evolutionary distances. By the end of the term, students had significantly increased their confidence in their ability to analyze all aspects of phylogenetic trees (*p* < 0.001).

#### Tutorials

STAMP was an essential component of the curriculum and was central for many of the student data analysis and visualization learning outcomes. We wanted to find out which learning activities the students found to be the most helpful in preparing them to use and interpret data in STAMP. Students reported that tutorials we created were useful, but perhaps unsurprisingly, it was actual use of the program and discussing it with the instructional staff that the students found to be essential (Table 3). All tutorials are publicly available at https://sites.google.com/g.ucla.edu/pumaa/home.

**TABLE 3:** Ranked usefulness of STAMP learning activities.

STAMP learning activity	Average score on five-point Likert Scale (*N* = 131)
Hands-on use of the program	4.5
One-on-one discussions with instructional staff	4.3
Tutorials (documents and videos)	3.6
Reading/reading assessment of STAMP user guide or articles	3.0

#### Statistical Reasoning and Conceptual Gains Measured by the SRBCI and Open-Ended Survey Responses

**FIGURE 5:**
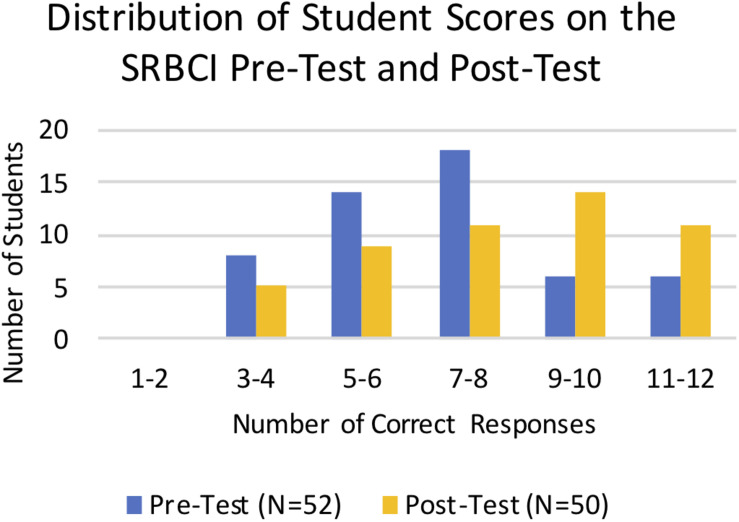
Distribution of Student scores on the SRBCI pre-test and post-test. The number of students plotted on the vertical axis is binned by the number of correct responses shown on the horizontal axis. The blue columns represent the pre-test scores and yellow columns represent the post-test scores. There was a significant increase in the SRBCI scores from the pre-test to the post-test (*p* = 0.01).

We used the SRBCI to directly assess student learning gains in core concepts related to repeatability of results, variations in data, hypotheses and predictions, and sample size. Students took the pre-test in the first week of the term and the post-test at the end of the term following the completion of all of the analysis modules. Scores for the pre-tests and post-tests were binned by number of correct responses and plotted to compare the overall distribution of scores (Figure 5). The distribution of the post-test scores is more skewed to the right, demonstrating overall improvement on the SRBCI for the combined cohorts. Statistical reasoning gains between the pre-test and post-test groups were assessed using a Mann–Whitney nonparametric test. There was a statistically significant increase in pre-test (Mean = 58.7%, Mean Rank = 44.3, *N* = 52) to post-test (Mean = 69.3%, Mean Rank = 59.0, *N* = 50) scores (*p* = 0.01) on the SRBCI.

**FIGURE 6:**
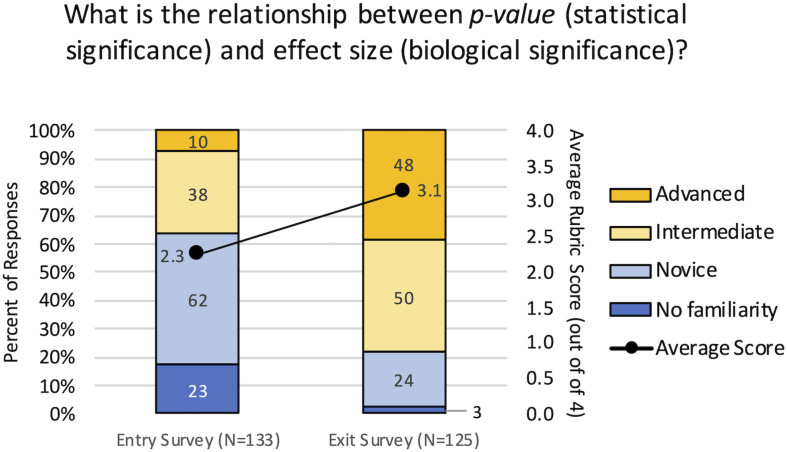
Conceptual gains in understanding the relationship between statistical and biological significance. Student responses to the open-ended question were evaluated using a rubric to assign them a level of competency from 1 = No familiarity, 2 = Novice, 3 = Intermediate, and 4 = Advanced. The primary axis indicates the percent of student responses demonstrating each level of competency for the entry and exit surveys. Blue indicates lower competency levels and yellow indicates higher competency levels. The secondary axis indicates the average score for all responses; there was a significant increase in the average score from the entry surveys to the exit surveys (*p* < 0.001).

A rubric-guided assessment of an open-ended survey question was used to determine whether the curricular interventions resulted in an increased understanding of the relationship between statistical significance (*p*-value) and biological significance (effect size). At the beginning of the term, 63.9% of students had no familiarity with the concept or held novice understanding, meaning the responses indicated they didn’t know, or they had multiple or complete misconceptions (Figure 6). By the end of the term, 78.4% of students held intermediate to advanced levels of understanding, and were able to demonstrate conceptual understanding of the relationship to varying degrees. The rubric scores from the Exit survey (Mean = 3.14, Mean Rank = 164.4, *N* = 125) were significantly higher than the Entry survey (Mean = 2.26, Mean Rank = 96.7, *N* = 135) by both the Mann–Whitney and *t*-tests (*p* < 0.001). These results demonstrate the shift from lower levels of competency to higher levels of competency in understanding the relationship between statistical and biological significance.

## Discussion

The increased availability of microbiome and other “big data” data sets has coincided with calls for life science undergraduates to have bioinformatics “minimum skill sets” or “core competencies” in order to meet the growing demand to analyze that data (Tan et al., 2009; Welch et al., 2016; Mulder et al., 2018; Sayres et al., 2018). PUMAA has been in use in the Research Immersion in Microbiology undergraduate laboratories at UCLA for a number of years, resulting in the development of a suite of instructional materials and tutorials to train students in many of the bioinformatics skills necessary to meet this demand. This curriculum focused on quantitative literacy, which is the intersection of critical thinking, math/statistics, and real-world contexts, and has been highlighted by the Association of American Colleges and Universities as an essential skill for undergraduates (Elrod, 2014). The PUMAA curriculum and associated analysis and visualization tools gave students opportunities to use multiple bioinformatic approaches to analyzing their data. Repeated practice with tools and integration of said tools into student-driven research projects increased self-reported confidence with data visualization and analysis. For example, use of STAMP enabled students to perform statistical tests on microbiome community and functional profiles, and improved their competence with statistical concepts such as statistical significance and biological significance. This was of particular interest due to the tendency of notice researchers to over interpret *p*-values and disregard the importance of effect sizes and confidence intervals (Nakagawa and Cuthill, 2007; Martínez-Abraín, 2008).

PUMAA presents a user-friendly, time-and-cost-effective approach to processing, analyzing, and visualizing marker gene microbiome data. It improves the accessibility and range of available microbiome investigations by providing users with a simple way to unify the output of various taxonomic identification platforms with a suite of tools for data analysis and visualization. The protocol accomplishes this by producing properly configured, formatted, and annotated files for analysis of taxonomic community profiles and inferred functional profiles. This process of data manipulation can often be performed by sequencing services for additional fees or completed by users with significant time commitment, both of which could be barriers for those with funding or time constraints. PUMAA is an open-source solution which is highly accessible to a wide spectrum of users, including undergraduates or other researchers interested in learning to conduct microbiome analyses, as it can be used as a GUI as well as a CLI. It provides an easy and flexible interface for a variety of users requiring a clear and brief interface for production of files needed for diversity analysis and data visualization for analysis of targeted amplicon sequencing studies. The demand for tools that meet this need is evidenced by the recent development of DNA metabarcoding data processing tools like the web-based SLIM (Dufresne et al., 2019) and minimal coding-required PEMA (Zafeiropoulos et al., 2020). Both of these tools produce OTU and/or ASV tables from raw metabarcode data that could be incorporated into the PUMA input pipeline for downstream data analysis and visualization.

In practice, the instructional staff runs the PUMAA program and provides students with files ready for use in Excel, ranacapa, STAMP, and other tools. One limitation of this approach is that students do not get direct experience with command-line bioinformatics, which is one of the core competencies for undergraduate life sciences education described by several different bioinformatics curriculum committees (Tan et al., 2009; Welch et al., 2016; Mulder et al., 2018; Sayres et al., 2018). However, the International Society for Computational Biology’s Curriculum Task Force has refined their core competencies and designated different user profiles requiring different *levels* of competency (Mulder et al., 2018). For example, an undergraduate in a 10-week microbial ecology course may be considered a “bioinformatics user,” rather than a “bioinformatics scientist” or “bioinformatics engineer,” and the steep learning curve required to gain CLI skills may not be practical with the limited time available. We focused instead on training students to perform all of the bioinformatic analyses needed for an authentic course-based undergraduate research experience in microbial ecology. PUMAA is not intended to replace comprehensive CLI tools such as QIIME or mothur, but rather serve as an entry point for novice researchers to analyze and visualize their datasets. Students that express interest in expanding their bioinformatics skills can be directed to a wealth of tutorials and resources for learning to code.

The PUMAA program and the curriculum described here have the potential to have a wide impact by making marker gene microbiome research accessible to researchers with multiple levels of experience, and with the included instructional module documents, it can be practically implemented in a classroom setting for undergraduates.

## Data Availability Statement

The datasets presented in this study can be found in online repositories. The names of the repository/repositories and accession number(s) can be found in the article/ Supplementary Material.

## Ethics Statement

The studies involving human participants were reviewed and approved by the UCLA Institutional Review Board (UCLA IRB). The patients/participants provided their written informed consent to participate in this study.

## Author Contributions

KM designed the PUMAA program and wrote the manuscript. JR created instructional materials, designed assessments, collected and analyzed assessment data, and contributed to the manuscript. CD contributed to writing the program, created instructional materials, and contributed to the manuscript. CS collected and analyzed assessment data, and contributed to the manuscript. SM consulted on the program and contributed to the manuscript. AF created instructional materials, designed assessments, and contributed to the manuscript. JMP designed the curriculum, created assessments, conceptualized the PUMAA program, and wrote the manuscript. All authors have reviewed and approved the manuscript.

## Conflict of Interest

The authors declare that the research was conducted in the absence of any commercial or financial relationships that could be construed as a potential conflict of interest.

## Footnotes

1 https://piphillin.secondgenome.com/

2 https://docs.qiime2.org/2020.2/tutorials/moving-pictures/

## Supplementary Material

Supplementary Table 1 PUMMA_Input and Output files.

Supplementary File 1 PUMAA_Curriculum details.

Supplementary File 2 PUMAA_Surveys.

Supplementary File 3 PUMAA_Rubric_Revised.
