# Supplementary material for: PUMAA: A Platform for Accessible Microbiome Analysis in the Undergraduate Classroom
Source: Front Microbiol. 2020 Oct 6;11:584699. doi: 10.3389/fmicb.2020.584699 (PMC7573227; doi:10.3389/fmicb.2020.584699)
Supplement: Supplementary Table 1 — PUMMA_Input and Output files. [file Table_1.DOCX]

Supplementary Material

# Supplementary Figures and Tables

## Supplementary Table 1. PUMAA Input and Output files.

| **PUMAA Taxonomy Input Files** | |
| --- | --- |
| MrDNA | 1. Taxonomy table output file “*.pr.fasta.otus.fa.OTU.txt” 2. FASTA sequences output file “*pr.fasta.otus.fasta” 3. Metadata table as *.txt tab-separated text file |
| Anacapa | 1. Taxonomy table output file “16S_ASV_raw_taxonomy_70.tsv” 2. FASTA sequence file(s) 3. Metadata table as *.txt tab-separated text file |
| QIIME | 1. OTU Table QIIME2 artifact file “*.qza” 2. Taxonomy QIIME2 artifact file “*.qza” 3. Representative sequences QIIME2 artifact file “*.qza” 4. Metadata table as *.txt tab-separated text file |
| **PUMAA Inferred Function Input Files** | |
| Piphillin | (These files are automatically output by PUMAA Taxonomy) 1. Piphillin OTU table as comma delimited text file “piphillinotu.csv”  2. Piphillin sequences file as FASTA file “piphillinseqs.fasta” |
| **PUMAA Taxonomy Output Files** | |
| Microsoft Excel | 1. Taxonomy table as *.tsv delimited text file. |
| ranacapa | 1. Taxonomy table as *.txt tab-separated taxonomy table; ranacapa requires that a taxonomy column be present with a header named “sum.taxonomy” and that the taxonomy hierarchy associated with each OTU or ASV is in a semicolon separated list. 2. Metadata as a delimited *.txt file, with the first column of the metadata file containing sample names that match the taxonomy table. |
| STAMP | 1. Taxonomy table as tab-separated *.tsv file with strict hierarchical and profile formatting restraints. Many classification tools that produce taxonomies pathways are not strictly hierarchical due to labeling or other errors. 2. Metadata as tab-separated *.tsv file. |
| QIIME | 1. Taxonomy table as .biom format table |
| Cytoscape | 1. Produces *.tsv/*.csv file where the first column is a sample, the second column is the taxonomic identification, and the third column is the taxonomic count or “weight”. The following columns for each row are the metadata features for the sample in the first column. |
| **PUMAA Inferred Function Output Files** | |
| Microsoft Excel | 1. Functional profile table as *.tsv delimited text file. |
| STAMP | 1. Functional profile table as tab-separated *.tsv file with strict hierarchical and profile formatting restraints. Many classification tools that produce functional pathways are not strictly hierarchical due to labeling or other errors. 2. Metadata as tab-separated *.tsv file. |
| Cytoscape | 1. Produces *.tsv/*.csv file where the first column is a sample, the second column is the functional identification, and the third column is the functional count or “weight”. The following columns for each row are the metadata features for the sample in the first column. |
