# Supplementary material for: PUMAA: A Platform for Accessible Microbiome Analysis in the Undergraduate Classroom
Source: Front Microbiol. 2020 Oct 6;11:584699. doi: 10.3389/fmicb.2020.584699 (PMC7573227; doi:10.3389/fmicb.2020.584699)
Supplement: Supplementary File 1 — PUMAA_Curriculum details. [file Data_Sheet_1.PDF]

MIMG 109BL/PUMA – Learning Objectives, Activities and Tutorials

CORE CONCEPT MODULE: Phylogenetic Trees

Adapted from "I, Microbiologist" Units 6.1, 6.2, 6.4, 7.1 and 7.2

Learning Objectives

- Explain the underlying algorithm(s) employed in sequence alignment (e.g. BLAST).
- Determine the two most related and two least related organisms from a short list of 16S rRNA sequences.
- Modify software parameters to achieve biologically meaningful results.
- Explain how data are organized in relational databases (e.g. NCBI).
- Understand that there is a probability of finding a given sequence similarity score by chance (the *P* value) and that the size of the target database affects the probability that you will see a particular score in a particular search (the E-value).
- Draw inferences about evolutionary relatedness of organisms based on phylogenetic trees using branch order and branch lengths.
- Explain the statistical support within a phylogenetic tree (bootstrap values).

Learning Activities and Tutorials

Summary: Students learn the theory behind pair-wise and multiple-sequence alignments and apply that knowledge to use BLAST and phylogenetic trees to identify bacterial isolates.
Overall step-by-step tutorial: Isolate Identification-Phylogenetic Trees Tutorial document

- Learn theory behind pair-wise sequence alignment and how to interpret BLAST results.
    - Presentation Slides: I-Micro slides_Alignments-BLAST_units6-1_6-2
- Inspect and trim the sequence reads to remove low quality base calls.
    - Video Tutorial: Trimming Sanger Sequences
- Find sequence matches for each isolate from public databases (e.g. NCBI BLASTn searches)
- Construct a phylogenetic tree with proper statistical support and four-taxon relationships.
    - Presentation Slides: I-Micro slides_units6-4_7-1_7-2
    - Video Tutorial: Multiple Sequence Alignment
    - Video Tutorial: Phylogenetic Tree Construction

CORE CONCEPT MODULE: Community Profile Analysis

Learning Objectives

- Describe the pipeline of metagenomic sequence processing.
- Describe the various sequence formats used to store DNA and protein sequences (e.g. FASTA, FASTQ).
- Compare and contrast operational taxonomic units (OTUs) and amplicon sequence variants (ASVs).
- Describe the components of an OTU or ASV abundance table.
- Store and interrogate datasets using spreadsheets or delimited text files.

- Evaluate different community profile visualization graphs.

Learning Activities and Tutorials

Summary: Students learn how the samples they collected were processed into the taxonomic abundance table they received. They then familiarize themselves with the breadth of the data by viewing the abundance table as an Excel sheet, followed by creating simple semi-quantitative data visualization plots at different taxonomic levels using Excel pivot tables.

- Learn theory behind the metagenomic 16S sequence processing pipeline.
  - Presentation Slides: 16S Sequence Data Processing
- Create community profile visualizations of OTU or ASV abundance using Excel pivot tables.
  - Video Tutorial: Microbiome Analyses and the Taxon Abundance Table Overview
  - Presentation Slides: Community Composition Exploration in Microsoft Excel_PivotTables

CORE CONCEPT MODULE: Microbial Diversity

Learning Objectives

- Define various parameters of diversity (e.g. richness, evenness).
- Compare various indices of diversity (e.g. alpha-diversity and beta-diversity).
- Define metadata and describe its role in analysis.
- Analyze microbial diversity data about samples from different environments.
- Interpret box and whisker plots, ordination plots (e.g. Principal Coordinate Analyses), and hierarchical clustering plots.
- Hypothesize about the relationship of diversity to other parameters (functional diversity, abiotic factors, plants present, etc.).

Learning Activities and Tutorials

Summary: The students learn about the theory behind different diversity statistics, and also create metadata tables to categorize the samples to be analyzed. They then use the tool ranacapa to explore the diversity of their samples.

- Learn theory behind alpha and beta diversity statistics.
  - Presentation Slides: Alpha and Beta Diversity Statistics
- eDNA data exploration using ranacapa
  - Embedded ranacapa explanations (https://gauravsk.shinyapps.io/ranacapa/)

CORE CONCEPT MODULE: Statistical Analysis of Taxonomic Profiles

Learning Objectives

- Develop testable hypotheses about metagenomic datasets.
- Analyze metagenomic datasets using statistical software.
- Explain the difference between statistical significance and biological significance.

- Define the following key statistics terms: effect size, confidence interval, p-value.
- Analyze microbial composition data about samples from different environments.
- Interpret heat maps, ordination plots (e.g. Principal Coordinate Analyses), and extended error bar plot, and other data visualizations.

Learning Activities and Tutorials

Summary: Students learn the theory of the Statistical Analysis of Metagenomic Profiles program through reading the STAMP Users Guide and the original article describing the program, and using them to answer reading assessment questions. Students then practice using the program through a guided tutorial.

- Learn the theory behind statistical analysis of taxonomic profiles
    - Reading Assignments: STAMP Users Guide and the article describing STAMP (Parks and Beiko, 2010)
    - Reading Assessment: "PUMA Reading Assessment_STAMP"
- Practice using STAMP to explore the sample data and test research hypotheses.
    - Video Tutorial: Statistical Analysis of Metagenomic Profiles (STAMP)
    - Presentation slides: PUMA STAMP Analysis

CORE CONCEPT MODULE: Inferred Functional Profiles

Learning Objectives

- Describe the workflow from 16S rRNA gene sequencing to functional community profile.
- Explain the limitations of PICRUSt/Piphillin analysis.
- Describe the KEGG databases.
- Connect inferred or predicted functional community analysis with taxonomic community analysis.
- Synthesize cultivation-dependent functions of characterized isolates with metagenomic functional capabilities.

Learning Activities and Tutorials

Summary: Students related inferred (predicted) functional profiles back to isolate characterization experiments in the first term. For example, if students identified isolates that produced antibiotics, they then assessed the level of known antibiotic production genes in their sample. Students can use any of the tools they have learned to analyze the functional profiles.

- Learn the theory behind inferring or predicting functional microbiome profiles from taxonomic abundance data.
    - Presentation Slides: Functional Profile Analysis with Piphillin and KEGG
- Analyze the inferred functional abundance tables using STAMP and optional advanced analysis tools.

ADVANCED ANALYSIS MODULE 6: Supplemental Tools

Learning Activities and Tutorials

- Students were encouraged, but not required, to perform advanced analyses on their datasets using one of the tools tested by the instructional staff, or a new tool that the students identified.
  - Presentation Slides: PUMA Supplementary Tools
- Cytoscape
  - https://cytoscape.org/
  - Written Tutorial: Cytoscape Setup Instructions and Tutorial
- QIIME2
  - https://qiime2.org/
  - Written Tutorial: QIIME 2 Setup Instructions and Tutorial
