# Supplementary material for: PUMAA: A Platform for Accessible Microbiome Analysis in the Undergraduate Classroom
Source: Front Microbiol. 2020 Oct 6;11:584699. doi: 10.3389/fmicb.2020.584699 (PMC7573227; doi:10.3389/fmicb.2020.584699)
Supplement: Supplementary File 2 — PUMAA_Surveys. [file Data_Sheet_2.PDF]

Entry Survey

Thank you for taking the time to complete this survey. Your responses will help the instructional staff assess your prior knowledge and evaluate the effectiveness of the 109BL curriculum.

Participation in this survey is worth 5 points. Individual completion of the survey will be recorded, but identifying information will not be associated with survey responses.

Page 1

1. * Please indicate your level of agreement or disagreement with the following statements.

	Strongly disagree	Disagree	No opinion or uncertain	Agree	Strongly agree
I am confident in my ability to interpret data sets and communicate those interpretations.	○	○	○	○	○
I am confident in my ability to make statistical inferences from data sets.	○	○	○	○	○
I am confident in my ability to extract relevant information from large data sets.	○	○	○	○	○
I am confident in my ability to make inferences about natural phenomenon using mathematical models.	○	○	○	○	○
I am confident in my ability to apply principles of logic (including the distinction between cause/effect and association) to problem solving.	○	○	○	○	○
I am confident in my ability to do quantitative work/data analysis.	○	○	○	○	○
I enjoy quantitative work/data analysis.	○	○	○	○	○
The idea of mathematics and statistics makes me anxious.	○	○	○	○	○
Mathematics, statistics, and computation are relevant to the life sciences.	○	○	○	○	○

Page 2

2. * How well do you understand...

	Not at all	Very little	Fairly well	Quite well	Very well
how to assess the quality of BLAST results?	○	○	○	○	○
how to assess the quality of a phylogenetic tree?	○	○	○	○	○
how to assess the quality of a statistical analysis or correlation?	○	○	○	○	○
the advantages and limitations of various statistical tests (e.g. Do you know when to use a T-test over a one-way ANOVA)?	○	○	○	○	○
how to design a computation-based solution to a hypothesis or research question (i.e. Do you know how to supplement your research with quantitative analysis)?	○	○	○	○	○
how to use bioinformatics databases (e.g. GenBank, etc.)?	○	○	○	○	○
how to graphically represent high volume data?	○	○	○	○	○
how to identify patterns in data?	○	○	○	○	○

Page 3

3. * How confident are you in your ability to analyze the following types of data plots?

	Not at all/I don't know what this is	Not very	Fairly confident	Quite confident	Very confident
Bar plot	○	○	○	○	○
Box and whisker plot	○	○	○	○	○
Heat map	○	○	○	○	○

Principle component analyses	◉	○	○	○	○	○
Scatter plots	◉	○	○	○	○	○
Histogram	◉	○	○	○	○	○
Extended error bar plots	◉	○	○	○	○	○

4. * **How confident are you in your ability to interpret the following aspects of phylogenetic trees?**

		Not at all/I don't know what this is	Not very	Fairly confident	Quite confident	Very confident
Bootstrap (resampling) values	◉	○	○	○	○	○
Topology (branching order)	◉	○	○	○	○	○
Evolutionary distances (branch lengths)	◉	○	○	○	○	○

Page 4

5. * **You are offered a position in a collaborative research project trying to find clinical and agricultural insights using microbial ecology data. You are given access to a database with information on the antibiotic production, antibiotic resistance, plant-growth promotion, and bioremediation abilities of rhizobacteria and their associated plants all over the world.**

- **What questions could you propose given such a large data set?**
- **How would you go about testing those questions and hypotheses?**

Paragraph

Path: p

6. * **What is the relationship between p-value and effect size?**

Paragraph

Path: p

7. * **Define, compare, and contrast the terms causation and correlation with consideration to the scope of this class.**

Paragraph

Path: p

Page 5

8. * **What experience do you hold, if any, in the intersections of biology and mathematics? Describe any computational analysis you've had to perform as part of a biology course at UCLA, a research project in which you participate, or anywhere else you deem relevant.**

Paragraph

Path: p

9. * **Given your experience in 109AL, what kinds of computational or quantitative analysis do you foresee yourself performing in BL?**

Paragraph

Path: p

10. * **In terms of computational and quantitative analysis, what insights do you expect to gain from this course? In other words, what would you like to learn about?**

Paragraph

Path: p

Exit Survey

Thank you for taking the time to complete this survey. Your responses will help the instructional staff assess student learning gains and evaluate the effectiveness of the 109BL curriculum.

Participation in this survey is worth 10 points. Individual completion of the survey will be recorded, but identifying information will not be associated with survey responses.

Page 1

1. * Please assess the quality of each of the following tutorials

	Don't remember	Not useful	Somewhat useful	Very useful	Essential
T1_Chromatograms					
T2_Multiple Sequence Alignments in MEGA					
T3_How to make a Phylogenetic Tree					
T4_Basic Community Profile Analysis Using Excel (OTU Table Exploration)					
T5_An Introduction to STAMP Bioinformatics Software					
T6_Background Info on Community Profile Data					
T7_Functional Community Analysis					

2. * How useful were each of the following for preparing you to use and interpret data in STAMP?

	Don't know; don't remember	Not useful	Somewhat useful	Very useful	Essential
Tutorials					
Reading/reading assessment of STAMP User Guide					
Reading/reading assessment of assigned STAMP articles					
One-on-one discussions with instructional staff					
Hands-on use of the program					

Page 2

3. * Please indicate your level of agreement or disagreement with the following statements.

	Strongly disagree	Disagree	No opinion or uncertain	Agree	Strongly agree
I understand how to construct a phylogenetic tree.					
I understand how to analyze a microbial community profile.					
I am confident in my ability to interpret data sets and communicate those interpretations.					
I am confident in my ability to extract relevant information from large data sets.					
I am confident in my ability to do quantitative analysis.					
I enjoy quantitative work/data analysis.					
The idea of mathematics and statistics makes me anxious.					
I am confident in my ability to use STAMP to analyze a taxonomic community profile.					
I am confident in my ability to make statistical inferences from data sets.					
I am confident in my ability to use STAMP to analyze a functional community profile.					
I understand how to graphically represent high volume data sets.					

I am confident in my ability to apply principles of logic (including the distinction between cause/effect and association) to problem solving.					
Mathematics, statistics, and computation are relevant to the life sciences.					

Page 3

4. * How well do you understand...

	Not at all	Very little	Fairly well	Quite well	Very well
how to assess the quality of BLAST results?					
how to assess the quality of a phylogenetic tree?					
how to find and use insightful phylogenetic anchors?					
how to assess the quality of a statistical analysis or correlation?					
the advantages and limitations of various statistical tests (e.g. Do you know when to use a T-test over a one-way ANOVA)?					
the advantages and limitations of each bioinformatics tool we used this quarter?					
how to interpret a STAMP-generated plot and integrate it into the research narrative?					
how to design a computation-based solution to a hypothesis or research question (i.e. Do you know how to supplement your research with quantitative analysis)?					
how to use bioinformatics databases (e.g. GenBank, RDP, etc.)?					
how to graphically represent high volume data?					
how to identify patterns in data?					

Page 4

5. * How confident are you in your ability to analyze the following types of data plots?

	Not at all/I don't know what this is	Not very	Fairly confident	Quite confident	Very confident
Bar plot					
Box and whisker plot					
Heat map					
Principle component analyses					
Scatter plots					
Histogram					
Extended error bar plots					

6. * How confident are you in your ability to interpret the following aspects of phylogenetic trees?

	Not at all/I don't know what this is	Not very	Fairly confident	Quite confident	Very confident
Bootstrap (resampling) values					
Topology (branching order)					
Evolutionary distances (branch lengths)					

Page 5

7. * You are offered a position in a collaborative research project trying to find clinical and agricultural insights using microbial ecology data. You are given access to a database with information on the antibiotic production, antibiotic resistance, plant-growth promotion, and bioremediation abilities of rhizobacteria and their associated plants all over the world.

- What questions could you propose given such a large data set?
- How would you go about testing those questions and hypotheses?

Paragraph

Path: p

8. * What is the relationship between p-value and effect size?

Paragraph

Path: p

Page 6

9. * How has this class changed your perception of the relationship between biology and mathematics/computation?

Paragraph

Path: p

10. * How do you perceive your own growth in terms of computational analysis in biology as a result of having taken this course?

Paragraph

Path: p

Page 7

11. * **What are the most significant concepts, techniques, and/or skills have you learned and gained as a result of taking this course?**

Paragraph

Path: p

12. * **Please explain what aspects of 109BL were the most valuable to you in gaining those concepts, techniques, and/or skills.**

Paragraph

Path: p

Close this window
