# Supplementary material for: PUMAA: A Platform for Accessible Microbiome Analysis in the Undergraduate Classroom
Source: Front Microbiol. 2020 Oct 6;11:584699. doi: 10.3389/fmicb.2020.584699 (PMC7573227; doi:10.3389/fmicb.2020.584699)
Supplement: Supplementary File 3 — PUMAA_Rubric_Revised. [file Data_Sheet_3.PDF]

PUMAA: Open-Ended Assessment Response Rubric

Criterion	Level of Proficiency			
	**No familiarity (1)**	**Novice (2)**	**Intermediate (3)**	**High (4)**
What is the relationship between p-value (statistical significance) and effect size (biological significance)?	Response indicates that they are **not familiar** with the concept; or **complete misconception**.	Response makes **some attempt to explain concepts or relationship** but **misconceptions are clear.**	Response demonstrates **some misconceptions** in their applications to testing statistical hypotheses, indicates **some understanding of the relationship**.	Response demonstrates **conceptual understanding of the relationship** between the terms.
Example student response	"I don't know."	"p-value indicates the accuracy of the statistical analysis of a set of data and its relation to effect size correlates to its accuracy of biological set data"	"P-value is used to determine if the difference between group is significant while the effect size is used to determine the size of the difference between groups."	"Just because something is statistically significant does not mean it is biologically relevant. One cause p-values to filter out first and then check effect sizes and CI to determine biological significance."

No Answer (blank) not scored.
